# Cognitive Distortions Associated with Loneliness: An Exploratory Study

**DOI:** 10.3390/bs15081061

**Published:** 2025-08-05

**Authors:** Kory Floyd, Colter D. Ray, Josephine K. Boumis

**Affiliations:** 1 Department of Communication, University of Arizona, Tucson, AZ 85721, USA; 2 Department of Communication, University of Tampa, Tampa, FL 33606, USA; cray@ut.edu; 3 Department of Communication Studies, University of South Florida, Tampa, FL 33620, USA

**Keywords:** loneliness, cognitive distortions, rational emotive behavior therapy

## Abstract

Loneliness is a significant challenge for millions worldwide, with chronic loneliness having harmful effects on physical health, mental well-being, and relationships. Cognitive distortions play an important role in perpetuating loneliness. Psychological interventions targeting such distortions have been effective at alleviating feelings of loneliness. However, less is known about which cognitive distortions are most prevalent among lonely individuals and how these distortions relate to loneliness and mental well-being. This exploratory study prescreened a Census-matched sample of 1000 U.S. adults for loneliness, then asked those in the top quartile (*N* = 237) to rate multiple patterns of cognitive distortion related to loneliness. Factor analyses identified six common and influential patterns of cognitive distortion (mindreading, future reward, catastrophizing, essentializing, deservedness, and externalizing). Essentializing was the most strongly endorsed factor, followed by mindreading and catastrophizing. Essentializing also evidenced the strongest correlation with loneliness. Additionally, the relationship between loneliness and participants’ stress was completely mediated by mindreading, catastrophizing, and essentializing. These findings highlight the importance of targeting specific cognitive distortions in loneliness interventions to effectively improve the mental well-being of lonely individuals.

## 1. Cognitive Distortions Associated with Loneliness: An Exploratory Study

Recent data shows that millions of adults continue to suffer from loneliness (Surkalim et al., 2022). Although some experiences of loneliness are temporary (J. T. Cacioppo et al., 2006), those who experience chronic loneliness often also experience several detriments to their physical health, mental well-being, and close relationships (see meta-analyses by Park et al., 2020; J. T. Cacioppo & Hawkley, 2009). As such, resolving chronic loneliness can result in a greater quantity and quality of meaningful relationships and also lead to significant improvement in one’s mental and physical health.

Prior research has shown that cognitive distortions perpetuate loneliness, and accordingly, the most consistently effective loneliness interventions are those that involve therapies that identify, challenge, and modify these forms of distorted thinking (J. T. Cacioppo et al., 2015; Hickin et al., 2021; Jarvis et al., 2019). However, there is no empirical data showing which specific cognitive distortions are most prevalent among highly lonely individuals and the extent to which specific cognitive distortions relate to loneliness. Therefore, identifying the most common and influential cognitive distortions that perpetuate their loneliness will allow those developing and implementing loneliness interventions to refine their efforts to more accurately target specific cognitive distortions.

Thus, the goals of this study are threefold. First, we aim to discover which specific cognitive distortions are most strongly endorsed amongst lonely individuals. Second, we will establish the extent to which specific cognitive distortions are associated with self-reports of loneliness. Finally, we will determine whether specific cognitive distortions mediate the relationship between loneliness and one related aspect of mental well-being, perceived stress (DeBerard & Kleinknecht, 1995; Glaser et al., 1985). We begin by summarizing the severity of the loneliness epidemic, both in terms of loneliness prevalence and the several physical and mental health issues that result from loneliness. We then consider the role that cognitive distortions play in perpetuating loneliness, providing a review of prior literature on the efficacy of loneliness interventions that target cognitive distortions and offering our own research questions regarding specific cognitive distortions that may be implicated in the experience of loneliness.

### 1.1. Loneliness

Loneliness is the aversive psychological state that exists when people recognize a discrepancy between their desired and achieved levels of meaningful social connection (J. T. Cacioppo et al., 2006). Although loneliness can negatively affect individuals at any stage of life, certain age groups are more vulnerable than others. Specifically, adolescents and young adults (Qualter et al., 2015; Victor & Yang, 2012), as well as the elderly (Cudjoe et al., 2020), tend to be the most afflicted. A meta-analysis by Surkalim et al. (2022) reported that global prevalence rates for loneliness range from 9.2 to 14.4 percent for adolescents and up to 24.2 percent for adults.

Episodic loneliness is common—such as when people are new to a city or workplace—and can even be adaptive insofar as it incentivizes the formation of new social relationships (J. T. Cacioppo et al., 2006). On the contrary, chronic loneliness is associated with multiple detriments to physical and mental well-being. Lonely individuals experience elevated pain (Jaremka et al., 2013) and increased risks of coronary heart disease (Thurston & Kubzansky, 2009), cardiovascular disease (Paul et al., 2021), stroke (Valtorta et al., 2016), and type 2 diabetes (Hackett et al., 2020). In addition, loneliness is a risk factor for both suicide ideation (Goldsmith et al., 2002) and attempted suicide (Stickley & Koyanagi, 2016) and a significant predictor of all-cause mortality (Holt-Lunstad et al., 2015; Luo et al., 2012; Perissinotto et al., 2012). Holt-Lunstad et al.’s (2015) meta-analytic review, which included data from over 3.4 million participants, reported that loneliness elevates the risk of premature mortality by 26 percent, even when other risk factors are controlled.

Chronic loneliness is also comorbid with a range of psychopathologies, including major depressive disorder (Erzen & Çikrikci, 2018), social anxiety disorder (Oren-Yagoda et al., 2022), eating disorders (Richardson et al., 2017), substance abuse disorders (Ingram et al., 2020), sleep disorders (Hom et al., 2020), personality disorders (Liebke et al., 2016), and dementias (Sundström et al., 2020). Lonely individuals are also more stressed (Richardson et al., 2017), less psychologically resilient (Gerino et al., 2017), and less compassionate toward those who are suffering (Floyd et al., 2023) compared to their non-lonely counterparts. Cross-sectional research by Meltzer et al. (2013) reported that the likelihood of loneliness is eight times higher in those with a diagnosed psychopathology, whereas the odds of loneliness are increased 20-fold for individuals with two or more diagnoses. It is therefore understandable that loneliness has been declared a public health crisis in the United States (Gerst-Emerson & Jayawardhana, 2015; National Academies of Sciences, Engineering, and Medicine, 2020) and elsewhere in the world (Dahl, 2020; DiJulio et al., 2018).

### 1.2. Cognitive Distortions in the Maintenance of Loneliness

Cognitive distortions are irrational or exaggerated thought patterns that are often instrumental in the onset or maintenance of psychopathologies. Originally described by Beck (1967) in the context of depression, cognitive distortions are information-processing patterns that lead to identifiable errors in thinking. Beck’s cognitive model claims that biased, distorted patterns of information processing are key contributors to the onset and persistence of psychopathological states. As articulated by Beck and others (e.g., Burns, 1980; Freeman & Oster, 1999), common cognitive distortion patterns include catastrophizing (believing that the worst possible outcome is likely, if not ensured); disqualifying the positive (discounting or ignoring positive experiences in favor of the negative); essentializing (using essentializing or derogatory statements to describe the self); mindreading (believing that one knows what others think about the self in the absence of evidence); and emotional reasoning (forming conclusions based on emotion rather than more valid data).

**Figure 1:**
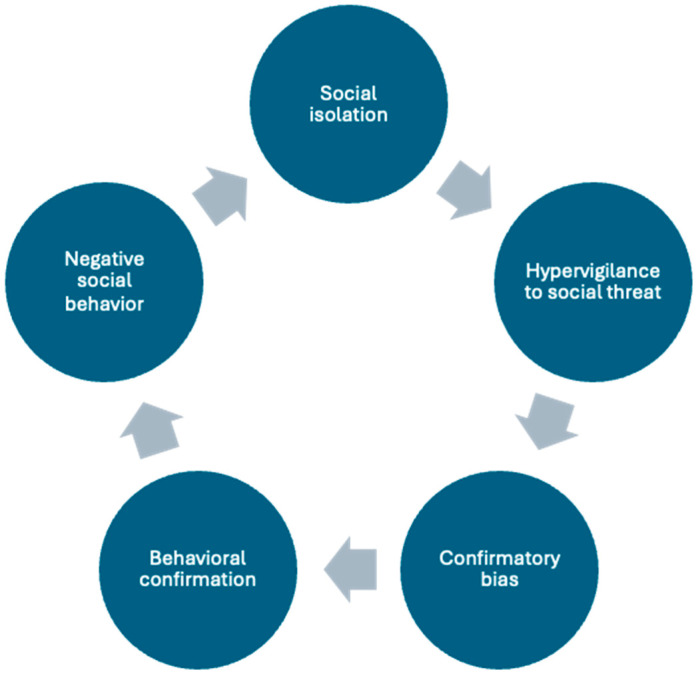
Regulatory loop maintaining loneliness, adapted from J. T. Cacioppo and Hawkley (2009, p. 451).

Distorted thinking plays a central role in maintaining loneliness. Cacioppo has argued that loneliness is generated and maintained via a regulatory loop in which the perception of social isolation activates hypervigilance for social threats, such as threats of ostracism or negative evaluations by others (J. T. Cacioppo & Hawkley, 2009). Such threats trigger dysfunctional thinking in the form of confirmation biases—interpreting ambiguous stimuli as confirming the perception that one is threatened—and biases in attention and memory (S. Cacioppo et al., 2016). These lead to behavioral confirmation processes in which individuals, guided by their biased perceptions, enact defensive, pessimistic, or even hostile interpersonal behavior, which understandably elicits negative cognitive and behavioral responses from others that confirm their negative expectancies. Once bolstered, these negative expectations lead lonely individuals to behave in increasingly negative ways, which only further contributes to their social isolation. A graphic representation of this self-reinforcing loop appears in Figure 1.

### 1.3. Treatments Based on Challenging Cognitive Distortions

Meta-analyses by Eccles and Qualter (2020) and Masi et al. (2011) both reported that psychological interventions show greater efficacy than alternatives, such as providing social support and offering social skills training. Masi et al. even confirmed that interventions designed to target cognitive distortions, specifically, were the most effective among all options examined. A 2021 meta-analysis by Hickin et al. added further support to the claim that psychological interventions for loneliness are effective, reporting an average effect size of *g* = 0.43 (95% CI = 0.18 to 0.68).

Many such interventions have taken a cognitive or cognitive–behavioral approach. Young (1982), for instance, proposed that chronically lonely individuals maintain patterns of beliefs and appraisals that facilitate their loneliness and isolation, such as beliefs that they deserve to be lonely or will be rejected if they attempt to form social bonds. Cognitive restructuring is therefore encouraged (see Clark, 2013), wherein distressed individuals are taught to identify, assess, and ultimately alter the distorted thinking patterns that are contributing to their distress (see Dobson & Dozois, 2010; Hollon & Dimidjian, 2009).

Multiple studies have tested interventions for loneliness that incorporated some aspects of identifying and modifying cognitive distortions. J. T. Cacioppo et al. (2015), for instance, assigned 688 U.S. soldiers to participate in an eight-session “social resilience training” intervention that addressed, among other topics, mindreading and the pitfalls of behavioral confirmation (wherein individuals attend to behavioral cues that confirm their distorted cognitions but ignore disconfirmatory cues). Compared to 450 soldiers who participated in an alternate training protocol, those who learned about social resilience reported significant reductions in loneliness. Similarly, Jarvis et al. (2019) recruited 828 residents from inner-city residential non-government care facilities in South Africa, who were randomly assigned either to a three-month intervention based on cognitive behavioral therapy or to a control group. The intervention offered psychoeducation about loneliness, the identification of individual cognitive distortions, and lessons on how to positively reframe distorted cognitions. As in J. T. Cacioppo et al.’s (2015) study, those in the intervention group reported significant reductions in loneliness (for additional examples, see Creswell et al., 2012; Jing et al., 2018; Käll et al., 2020; Kremers et al., 2006; Zhang et al., 2018).

### 1.4. The Present Study

Cognitive therapy, cognitive behavioral therapy, and cognitive restructuring have been effective at helping people (1) identify cognitive distortions that may be maintaining their loneliness, (2) assess the validity of such beliefs by examining their logic and looking for disconfirming evidence, and (3) reframe distorted cognitions to ones that are more functional. However, when it comes to those who are particularly lonely, it is still unclear which cognitive distortions are experienced most frequently and are most strongly related to loneliness.

Determining the most prevalent and influential cognitive distortions regarding loneliness is a consequential question for the development of loneliness interventions. Whereas one-on-one therapy will understandably identify and focus on the cognitive distortions that are most distressing to each individual client, interventions intended to have a broader reach would benefit from empirical evidence that shows which cognitive distortions are most prevalent and thus most important to target in large-scale interventions. In taking up this topic, the present exploratory study is guided by the following questions:

RQ1. Among lonely individuals, which forms of cognitive distortion related to loneliness are most prevalent?
RQ2. Among lonely individuals, which forms of cognitive distortion related to loneliness are most strongly correlated with the experience of loneliness?

Beyond identifying which distorted cognitive patterns are most common and most strongly related to loneliness, we also explored the question of which patterns might mediate the association between loneliness and negative outcomes. For instance, might distorted thinking patterns be a mechanism through which loneliness perpetuates negative states? If so, then interventions designed to target the most pronounced mechanisms could be expected to produce the most beneficial results with respect to those states. As an initial exploration of this topic, we chose the negative outcome of stress, which research has long shown to be robustly related to loneliness (see DeBerard & Kleinknecht, 1995; Glaser et al., 1985). Our exploration was guided by the following question:

RQ3. Among lonely individuals, which forms of cognitive distortion related to loneliness mediate the association between loneliness and stress?

## 2. Method

### 2.1. Loneliness Pretest

Prospective participants were prescreened for loneliness. Participants (*N* = 1000) were 490 men, 497 women, 2 transgender individuals, and 11 individuals who did not report a gender identity, ranging in age from 18 to 91 years old (*M* = 46.94 years, *SD* = 16.96). Participants were recruited using Prolific Academic and represented a Census-matched sample of U.S. American adults (U.S. Census Bureau, n.d.). Most (73.5%) identified as White, whereas 15.0% were Black/African American, 8.7% were Asian, 7.8% were Latino/a, 3.1% were Native American or Aleut, 0.7% were Middle Eastern/Northern African, 0.6% were Arab, 0.4% were Native Hawaiian/Pacific Islander, and 1.5% claimed other racial origins. Additionally, 11.3% of participants identified as Hispanic and 88.7% identified as non-Hispanic.

The study (including both the loneliness pretest and the full study) was approved by the institutional review board, and participants provided informed consent for their participation.

Each participant completed the 20-item Revised UCLA Loneliness Scale version 3 (Russell, 1996), which comprises 11 negatively worded items (e.g., “No one really knows me well”) and 9 positively worded (reverse-scored) items (e.g., “There are people I feel close to”). Participants responded to the items using a 4-point scale, with higher scores indicating greater levels of loneliness, and a total loneliness score was calculated by summing the individual scores; McDonald’s ω = 0.96. Participants were also informed that their results may qualify them for an additional study and were asked to indicate whether they would be interested in being invited to such a study.

Loneliness scores in the pretest ranged from 20.00 to 80.00 (*M* = 44.00, *SD* = 13.96). Loneliness did not vary as a function of gender, with women (*M* = 43.70, *SD* = 14.18) and men (*M* = 44.54, *SD* = 13.67) scoring nearly identical average scores, *t* < 1.^1^. Age was negatively associated with loneliness, *r* (994) = −0.29, *p* (two-tailed) < 0.001. Those reporting a Hispanic ethnicity were lonelier (*M* = 47.50, *SD* = 14.70) than those reporting a non-Hispanic ethnicity (*M* = 43.71, *SD* = 13.79), Welch’s *t* (135.95) = 2.71, *p* (two-tailed) = 0.01, Cohen’s *d* = 0.27. Participants who identified as Black/African American were lonelier (*M* = 47.57, *SD* = 14.09) than those who did not (*M* = 43.37, *SD* = 13.85), Welch’s *t* (203.22) = 3.41, *p* (two-tailed) < 0.001, Cohen’s *d* = 0.30. Participants who identified as Latino/a were lonelier (*M* = 48.88, *SD* = 15.05) than those who did not (*M* = 43.59, *SD* = 13.80), Welch’s *t* (88.33) = 3.00, *p* (two-tailed) = 0.003, Cohen’s *d* = 0.38. Participants who identified as White were less lonely (*M* = 43.39, *SD* = 14.16) than those who did not (*M* = 45.74, *SD* = 13.24), Welch’s *t* (485.44) = −2.34, *p* (two-tailed) = 0.016, Cohen’s *d* = 0.17.^2^ No other racial comparisons were significant.

Loneliness scores were divided into quartiles. Participants (1) whose scores were in the highest quartile, which comprised scores ranging from 54 to 80; (2) who passed an embedded attention check in the pretest; and (3) who indicated an interest in the subsequent study were invited to complete the full study on cognitive distortions. Women and men were equally likely to meet all the qualifications for the full study, χ^2^ (1) = 0.09, *p* = 0.76. Those who qualified were younger, on average (*M* = 41.30 years, *SD* = 14.46), than those who did not qualify (*M* = 48.88 years, *SD* = 17.33), Welch’s *t* (520.17) = 18.66, *p* (two-tailed) < 0.001, *d* = 0.48. Out of 254 invitations extended, 241 participants provided data for the full study, resulting in a response rate of 95 percent.

### 2.2. Full Study

#### 2.2.1. Participants

Data from a total of 241 participants were initially collected, but four participants did not satisfy both of the two embedded attention checks, leaving an *N* of 237. Of these 237 participants, 120 were assigned female at birth and 117 were assigned male at birth. Participants’ ages ranged from 18 to 78 years (*M* = 41.20 years, *SD* = 14.43). Participants’ current gender identities were cisgender woman (*n* = 64), cisgender man (*n* = 63), man (*n* = 59), woman (*n* = 51), non-binary (*n* = 8), questioning (*n* = 4), transgender man (*n* = 3), genderqueer (*n* = 2), agender (*n* = 2), and transgender woman (*n* = 1).^3^ With respect to ethnicity, 39 participants identified as Hispanic and 197 as non-Hispanic, with 1 failing to report. Participants’ racial identities were White (*n* = 172), Black/African American (*n* = 40), Asian (*n* = 24), Latino/a (*n* = 31), Native American or Aleut (*n* = 12), Arab (*n* = 4), Native Hawaiian/Pacific Islander (*n* = 3), Middle Eastern/Northern African (*n* = 2), or another racial identity (*n* = 4). With respect to education, 30.9% had a high school diploma or less, 9.3% had a vocational, technical, or trade school diploma, 13.1% had an associate’s degree, 33.1% had a bachelor’s degree, 9.7% had a master’s degree, and 3.8% had a doctoral degree. Participants represented 45 of 50 U.S. states. At the time of the study, an equal number of participants were married or partnered (*n* = 102) and single/unattached (*n* = 102), whereas 31 were divorced, 1 was widowed, and 1 did not report a relationship status.

#### 2.2.2. Procedure

Participants were recruited via Prolific Academic using a list of identification numbers that restricted invitations to those whose pretest data qualified them for the full study.

#### 2.2.3. Measurement

Cognitive distortions were assessed with 31 statements written to represent a range of dysfunctional thinking patterns (Burns, 1989, 1999; Freeman & DeWolf, 1992; Freeman & Lurie, 1994; Gilson & Freeman, 1999), from catastrophizing (“When I feel lonely, it is the worst thing in the world”), mindreading (“I know other people think poorly of me, even when they don’t tell me that”), and emotional reasoning (“How do I know that others reject me? I know because I feel it”) to overgeneralization (“Nobody wants to be emotionally connected to me, ever”), fallacy of fairness (“It isn’t fair that I am as lonely as I am”), and Heaven’s reward (“If I can just tolerate my loneliness now, I know I will be rewarded later”). The statements were written by a licensed clinical mental health counselor with both a master’s degree and a post-master’s professional degree in mental health counseling whose training emphasized rational emotive behavior therapy. Each item was measured on a Likert scale ranging from 1 (*strongly disagree*) to 7 (*strongly agree*). A full list of the items, along with each item’s correlation with participants’ loneliness scores, appears in Table 1.

Stress was measured with the 10-item Perceived Stress Scale (Cohen et al., 1983), which asks participants to consider their feelings and thoughts over the previous month and to indicate how often they felt upset, nervous, stressed, and unable to cope. Responses were measured on a scale of 1 (*never or almost never*) to 7 (*always or almost always*), and a score for stress was calculated by summing the individual scores, with higher scores indicating more perceived stress; McDonald’s ω = 0.89.

## 3. Results

### 3.1. Descriptive Statistics

Loneliness scores in the full study ranged from 54.00 to 80.00 (*M* = 62.63, *SD* = 6.37). Loneliness did not vary as a function of gender, with women (*M* = 62.56, *SD* = 6.64) and men (*M* = 62.71, *SD* = 6.12) scoring nearly identical average scores, *t* < 1. Age was not significantly associated with loneliness, *r* (235) = 0.08, *p* (two-tailed) = 0.21, and did not differ as a function of ethnicity. Participants who identified as Black/African American were lonelier (*M* = 64.63, *SD* = 6.50) than those who did not (*M* = 62.23, *SD* = 6.29), Welch’s *t* (54.85) = 2.14, *p* (two-tailed) = 0.037, Cohen’s *d* = 0.38. No other racial comparisons were significant.

Stress scores ranged from 13.00 to 68.00 (*M* = 45.87, *SD* = 11.74). Stress did not vary as a function of gender, with women (*M* = 47.01, *SD* = 11.70) and men (*M* = 44.70, *SD* = 11.72) scoring similarly, *t* < 1. Stress was inversely associated with age, *r* (235) = −0.20, *p* (two-tailed) < 0.001, but did not differ as a function of ethnicity. Participants who identified as White reported more stress (*M* = 46.94, *SD* = 11.12) than those who did not (*M* = 42.82, *SD* = 12.75), Welch’s *t* (104.65) = 2.31, *p* (two-tailed) = 0.023, Cohen’s *d* = 0.36. No other racial comparisons were significant.

### 3.2. Dimension Reduction

We subjected the 31 items from the cognitive distortions scale to a principal-components factor analysis with Oblimin rotation. The initial exploratory factor analysis (EFA) identified seven factors with eigenvalues exceeding 1, yet an examination of the factor loadings revealed that the seventh factor comprised only a single item. Thus, the EFA was constrained to identify six factors which explained 63.75 percent of cumulative variance, KMO = 0.90, Bartlett’s test of sphericity χ^2^ (465) = 3977.43, *p* < 0.001. To interpret the pattern matrix, we required primary loadings of ≥0.50 and secondary loadings of <0.30. Thirteen items loaded strongly on more than one factor and were therefore removed before the final EFA was conducted, resulting in a clean six-factor structure accounting for 74.63 percent of the variance.

The first factor, labeled **Mindreading**, included four items describing knowing that others reject the self, even without evidence. The second factor included two items proposing that if one can tolerate loneliness now, one will be rewarded in the future, and was labeled **Future Reward**. Four items comprised the third factor, which reflected the idea that loneliness is intolerable and the worst possible outcome, and was labeled **Catastrophizing**. The fourth factor consisted of three items expressing the sentiment that one was “simply the kind of person who tends to be lonely” and was labeled **Essentializing**. The fifth factor contained three items expressing the sentiments that “I deserve to be lonely” and “I am destined to be lonely,” and was labeled **Deservedness**. Finally, two items made up the sixth factor, which proposed that other people should change their behavior to be more inclusive and was labeled **Externalizing**.

Scores for each dimension were calculated by averaging the items in that dimension (higher scores indicated stronger endorsement). Factor loadings, internal reliabilities, and descriptive statistics appear in Table 2.

### 3.3. Research Questions

The first research question asked which forms of cognitive distortion related to loneliness are most strongly endorsed. To address the question, we entered the means of the six dimensions into a within-subjects analysis of variance. The comparison, which employed Huynh–Feldt-corrected degrees of freedom due to violation of compound symmetry assumptions, indicated significant differences among the six mean scores, *F* (4.11, 970.62) = 107.71, *p* < 0.001. Table 3 indicates the significant pairwise comparisons. As the table reflects, the most strongly endorsed pattern of dysfunctional thinking was essentializing, followed by mindreading and then by catastrophizing.

The second research question asked which forms of cognitive distortion related to loneliness are most strongly correlated with the experience of loneliness. To address the question, the six dimensions were entered into two-tailed Pearson correlations with participants’ loneliness scores. Of the six dimensions, essentializing showed the strongest association with loneliness, *r* (235) = 0.41, *p* < 0.001. The lonelier participants were, the more strongly they believed they were simply the kind of person who tends to be lonely. Mindreading, *r* (235) = 0.32, *p* < 0.001, and deservedness, *r* (235) = 0.31, *p* < 0.001, had nearly identical correlations with loneliness. The lonelier participants were, the more strongly they believed that they could tell that others rejected them, even without their saying so, and the more strongly they believed that they deserved to be lonely. Catastrophizing was moderately associated with loneliness, *r* (235) = 0.26, *p* < 0.001, as was future reward, *r* (235) = −0.25, *p* < 0.001. Thus, the lonelier participants were, the more intolerable they found loneliness to be, and the less likely they were to believe that their present loneliness would translate into future reward. Only externalizing showed a nonsignificant association with loneliness, *r* (235) = 0.07, *p* = 0.24, meaning that loneliness was unassociated with participants’ beliefs that others should change their behaviors to be more inclusive.

The third research question asked which patterns of distorted cognition related to loneliness mediate the association between loneliness and stress. Using Hayes’s PROCESS macro model 4, we tested the six forms of cognitive distortions as parallel mediators of the relationship between stress and loneliness. The direct effect of stress on loneliness was nonsignificant (*B* = 0.15, *p* = 0.20), and therefore any significant indirect effects are considered fully mediated. Of the six factors tested as mediators, three returned significant indirect effects. The relationship between stress and loneliness was fully mediated by mindreading (*B* = 0.14), catastrophizing (*B* = 0.07), and essentializing (*B* = 0.13). Mediation analyses appear in Table 4.

## 4. Discussion

Whereas episodic loneliness is common and can even be beneficial, chronic loneliness is associated with a wide range of mental, physical, and social detriments for the millions who suffer from it worldwide (J. T. Cacioppo & Hawkley, 2009; Park et al., 2020). Like many distressing psychological states, loneliness is maintained, in part, via cognitive distortions comprising irrational or exaggerated thought patterns (Beck, 1967; Burns, 1980; Freeman & Oster, 1999). Psychotherapies employing a cognitive or cognitive behavioral approach—which have shown promise for treating loneliness—focus heavily on identifying and challenging cognitive distortions with the goal of replacing them with more functional ways of thinking (J. T. Cacioppo et al., 2015; Hickin et al., 2021; Jarvis et al., 2019). Consequently, such therapies could benefit from evidence showing which patterns of cognitive distortion are most common and most strongly correlated with loneliness among the lonely, which was the goal of this exploratory project.

We assessed loneliness in a Census-matched sample of 1000 U.S. adults (U.S. Census Bureau, n.d.) and then invited participation from those whose loneliness scores were in the highest quartile, who passed attention checks, and who indicated an interest in participating. Those participants were assessed for their endorsement of a range of common cognitive distortions, which were later compared to their scores of loneliness and stress. Using EFA, we identified six patterns of cognitive distortion that reflected the lonely sample: mindreading, future reward, catastrophizing, essentializing, deservedness, and externalizing. Essentializing was the most strongly endorsed, followed by mindreading and then catastrophizing, and essentializing showed the strongest association with loneliness. We further found that the association between loneliness and stress was fully mediated by mindreading, catastrophizing, and essentializing.

Although exploratory in nature, these findings illuminate the nature of distorted thinking among lonely individuals in a manner not previously articulated empirically. Of all the ways in which lonely people can think dysfunctionally about their loneliness, essentializing—the idea that one is “simply the type of person who tends to be lonely”—emerged as not only the most strongly endorsed but also the most strongly associated with loneliness itself (*r* = 0.41). This suggests the possibility that part of what maintains an individual’s loneliness is the belief that “if I am lonely, that is because I am simply the type of person who tends to be lonely.” This may imply to the individual that loneliness is an essential aspect of their personhood, much like a physical characteristic or a personality trait. If an individual believes this, it is not a stretch to suggest that they may perceive such an aspect to be unchangeable, or at least, difficult to modify.

Mindreading—the belief that one knows that others reject them, even in the absence of evidence—was the second-most strongly endorsed pattern and the second-most strongly correlated with loneliness (*r* = 0.32). Individuals with this pattern of distorted thinking are inclined to believe that they already “know” that others do not want to befriend them or spend time with them, which can imply that efforts to create or strengthen social bonds are destined to fail and therefore not worth the effort. It is easy to see how such a thought pattern would contribute to the maintenance of loneliness by convincing lonely people that efforts to connect socially with others would be in vain. Deservedness (the idea that if one is lonely, it is because one deserves to be lonely), catastrophizing (the idea that loneliness is an intolerable state that one cannot abide), and future reward (the belief that one will be rewarded in the future for being lonely now) all showed moderate associations with loneliness.

We also examined the association between loneliness and stress, which is well established empirically (DeBerard & Kleinknecht, 1995; Glaser et al., 1985), to explore which cognitive distortions, if any, mediate that relationship. Once again, essentializing and mindreading showed the strongest effects, although catastrophizing was also a significant mediator. Although these data are cross-sectional and therefore cannot support causal inferences, the mediation findings are consistent with the possibility that loneliness is associated with stress, in part, *because* loneliness exacerbates distorted beliefs regarding essentializing, mindreading, and catastrophizing. In each case, the loneliness–stress association was fully mediated. Again, we cannot conclude from these cross-sectional findings that the mediation works in this fashion, but these findings are consistent with that possibility.

### 4.1. Implications

The primary implication of these findings relates to cognitive or cognitive–behavioral psychotherapies targeting loneliness. It is certainly the case that individual clients present with their own individual cognitive distortions that a clinician must then identify in order to challenge and treat. Nonetheless, cognitive and cognitive behavioral therapies also articulate particular patterns of distorted thinking that are common across presenting complaints, including catastrophizing, mindreading, overgeneralization, and emotional reasoning (Ellis & MacLaren, 2005; Sokol & Fox, 2019). This exploratory study goes further by identifying the cognitive distortions that are most common for people suffering from loneliness, specifically, as well as those most strongly associated with loneliness.

At the individual level, this information can assist therapists who are treating loneliness in targeting therapeutic efforts toward the most common distorted thinking patterns characterizing loneliness. Beyond that, this detail can inform broader-reaching interventions for loneliness, such as internet-based interventions (Kaiser et al., 2021), to teach lonely individuals to assess, challenge, and ultimately modify these distorted thinking patterns if they experience them. Such interventions could be developed and tested clinically in randomized controlled trials for their efficacy in reducing loneliness and even improving its detrimental correlates, such as depression, anxiety, and stress.

### 4.2. Liabilities and Strengths

When considering these applied implications, however, certain limitations of the study must be acknowledged. First, the sample was limited to adults in the United States. No generalization of these findings is warranted to children or to non-U.S. individuals, and additional research to establish the generalizability of the results beyond the present sample would certainly be justified. Second, the sample for the full study was not a clinical sample. We did not ask participants directly whether they were currently receiving psychotherapy for loneliness, or whether they had received such treatment in the past, but the sample was not purposively drawn from individuals in treatment. Consequently, although participants scored relatively highly on loneliness (*M* = 62.63 out of a possible score of 80.00), their loneliness may not have been causing them clinically significant distress. As such, it would be empirically useful to replicate the study with a clinical sample to increase confidence in the validity of the findings. Future studies should also consider including measures of anxiety, social isolation, and other relevant constructs.

Despite these limitations, this exploratory study also benefited from the use of a representative sample for prescreening loneliness. Some loneliness studies (e.g., Hyland et al., 2018; Özdemir & Tuncay, 2008) rely on convenience samples, which frequently restricts variance on age, education, and geographic diversity and oversamples some genders and racial and ethnic groups while undersampling others. In contrast, our initial sample was Census-matched demographically to the U.S. American adult population (U.S. Census Bureau, n.d.), ensuring a representative prescreening on loneliness from which the final sample was drawn. Of course, the final sample—because it was based on loneliness scores—was not a representative sample, yet it still evidenced better demographic and geographic diversity than many samples recruited by convenience alone.

## 5. Conclusions

Loneliness is a widespread condition that can be highly detrimental to well-being, making relevant any effective means of reducing it. Psychotherapies focused on cognitive restructuring aim to identify, challenge, and modify cognitive distortions, implying that those distortions that are most common for lonely people–and most strongly correlated with loneliness–are candidates for specific, focused therapeutic attention. This exploratory study identified six particular patterns of distorted cognition characteristic of lonely U.S. adults and found that essentializing—the belief that one is “simply the type of person who tends to be lonely”—was not only the most strongly endorsed but also the most strongly associated with loneliness.

## Figures and Tables

**Table 1:** Descriptive statistics and correlations with loneliness for study items (*N* = 237).

Item	*M*/*SD*	*r* with Loneliness
I feel that I am disconnected from others, and because I feel that way, it is therefore true.	4.42/1.66	0.38 **
I use my emotions to tell me what’s true about my relationships.	4.18/1.46	0.07
How do I know that others reject me? I know because I feel it.	4.18/1.75	0.27 **
Even when they don’t say so, I can tell other people don’t want to be emotionally connected to me.	4.35/1.70	0.31 **
I know other people think poorly of me, even when they don’t tell me that.	4.17/1.73	0.28 **
When I feel lonely, it is the worst thing in the world.	3.42/1.83	0.22 **
Feeling lonely is terrible and I cannot stand it.	3.82/1.78	0.24 **
Being lonely is the worst thing that could happen to me.	2.75/1.76	0.21 **
I cannot stand feeling lonely.	3.70/1.82	0.23 **
Other people need to change their behaviors so that I feel less lonely.	2.52/1.49	0.07
Other people should change how they interact with me so that I feel more connected.	2.59/1/61	0.06
Being lonely isn’t my fault; I am a victim of what happens around me.	3.02/1.45	0.14 *
I am lonely because of my circumstances.	5.00/1.71	0.24 **
I have no control over how lonely I am.	3.48/1.60	0.25 **
Nobody wants to be emotionally connected to me, ever.	3.36/1.80	0.46 **
Everyone I want to feel emotionally connected to rejects me.	3.34/1.72	0.34 **
I always feel rejected by others.	3.98/1.84	0.31 **
I had a hard time connecting with someone once. I will feel lonely forever.	2.93/1.72	0.23 **
In the past, I have had difficulty forming positive emotional connections. I am destined to feel lonely for the rest of my life.	3.78/1.88	0.32 **
Even though I have had good relationships before, I am destined to be lonely.	3.73/1.88	0.37 **
I’ve had some emotional connections with people in the past, but those don’t count; I am likely to be lonely forever.	3.12/1.74	0.31 **
It isn’t fair that I am as lonely as I am.	3.63/1.83	0.23 **
If life were more fair, I wouldn’t be so lonely.	3.65/1.86	0.26 **
If I can just tolerate my loneliness now, I know I will be rewarded later.	3.19/1.71	−0.23 **
If I bear my lonely feelings now, I’ll be rewarded in the future.	3.03/1.60	−0.23 **
If I am lonely, that must mean I deserve to be lonely.	3.07/1.84	0.27 **
I wouldn’t be lonely if I didn’t deserve to be.	3.00/1.77	0.21 **
I am simply the kind of person who tends to be lonely.	4.97/1.75	0.42 **
I’m just the type of person who is usually lonely.	4.71/1.81	0.40 **
Other people are either with me or against me.	3.39/1.81	0.19 **
In relationships, I am either a success or I’m a total failure.	3.80/1.72	0.09

*Notes.* * *p* < 0.05; ** *p* < 0.01 (two-tailed).

**Table 2:** Factor loadings, internal reliabilities, and descriptive statistics for seven identified factors.

Item	1	2	3	4	5	6
How do I know that others reject me? I know it because I feel it.	**0.77**	0.05	−0.07	0.02	−0.09	−0.07
Even when they don’t say so, I can tell other people don’t want to be emotionally connected to me.	**0.72**	0.12	−0.05	0.11	−0.15	0.14
I use my emotions to tell me what’s true about my relationships.	**0.65**	−0.19	−0.01	−0.06	0.16	−0.23
I know other people think poorly of me, even when they don’t tell me that.	**0.64**	0.15	−0.02	0.13	−0.25	0.22
If I can just tolerate my loneliness now, I know I will be rewarded later.	−0.02	**0.89**	0.05	−0.01	−0.02	−0.07
If I bear my lonely feelings now, I’ll be rewarded in the future.	0.06	**0.89**	−0.03	−0.02	0.11	−0.10
When I feel lonely, it is the worst feeling in the world.	0.03	−0.04	**−0.90**	−0.03	−0.05	0.03
I cannot stand feeling lonely.	0.02	−0.07	**−0.89**	0.08	0.06	0.04
Feeling lonely is terrible and I cannot stand it.	0.04	0.01	**−0.88**	0.09	0.05	0.01
Being lonely is the worst thing that could happen to me.	−0.05	0.07	**−0.82**	−0.10	−0.05	−0.09
I am simply the kind of person who tends to be lonely.	0.05	−0.07	0.02	**0.93**	0.06	0.02
I’m just the type of person who is usually lonely.	0.02	−0.01	−0.01	**0.88**	0.01	−0.03
I am lonely because of my circumstances.	−0.07	0.05	−0.05	**0.73**	−0.03	−0.04
I wouldn’t be lonely if I didn’t deserve to be.	0.01	−0.01	−0.04	−0.07	**−0.92**	−0.02
If I am lonely, that must mean I deserve to be lonely.	0.06	−0.01	−0.05	0.08	**−0.83**	0.01
I’ve had some emotional connections with people in the past, but those don’t count; I am likely to be lonely forever.	0.13	−0.18	0.02	0.18	**−0.54**	−0.27
Other people need to change their behaviors so that I feel less lonely.	−0.08	0.10	−0.08	0.03	−0.20	**−0.83**
Other people should change how they interact with me so that I feel more connected.	0.14	0.16	−0.04	0.09	0.06	**−0.79**
Internal Reliability (Alpha)	0.76	0.83	0.90	0.81	0.80	0.80
Mean	4.22	3.11	3.42	4.76	3.06	2.42
Standard Deviation	1.27	1.53	1.57	1.50	1.50	1.42

*Note*. Primary factor loadings are in bold type.

**Table 3:** Pairwise comparisons of dimensions of cognitive distortions about loneliness (*N* = 237).

Dimension	Mean
Mindreading	4.22 _a_
Future Reward	3.11 _b_
Catastrophizing	3.42 _c_
Essentializing	4.76 _d_
Deservedness	3.06 _b_
Externalizing	2.42 _e_

*Note*. Mean values with different subscripts differ significantly from each other, per two-tailed pairwise comparisons.

**Table 4:** Mediating effects of loneliness beliefs on association between loneliness and stress (*N* = 237).

Total Effect *B* (*p*)	Direct Effect *B* (*p*)	Relationship	Indirect Effect *B* [95% CI]
Stress → Loneliness	Stress → Loneliness	Stress → Mindreading → Loneliness	0.14 [0.05, 0.26]
0.60 (<0.001)	0.15 (0.20)	Stress → Future Reward → Loneliness	0.05 [−0.01, 0.13]
		Stress → Catastrophizing→ Loneliness	0.07 [0.01, 0.15]
		Stress → Essentializing→ Loneliness	0.13 [0.02, 0.26]
		Stress → Deservedness → Loneliness	0.04 [−0.05, 0.15]
		Stress → Externalizing → Loneliness	0.002 [−0.03, 0.03]
		Total Indirect Effect	0.45 [0.29, 0.62]

## Data Availability

Data are viewable at https://osf.io/d3yme/?view_only=e7d22dfdc3d24be7942f079c02e859d9.

## Notes

1 This comparison temporarily suppressed the data of two participants whose reported gender was other than female or male due to the small cell size.

2 Racial comparisons were conducted separately because participants could select more than one racial identity.

3 Cell sizes for gender identity and racial identity sum to >237 because participants could select multiple categories.
